# The Fabian Report on Professional Associations

**Published:** 1917-06-02

**Authors:** 


### MEDICAL ORGANISATION.

### The Fabian Report on Professional Associations.

This Report, issued as two special supplements to the *New Statesman* of April 21 and 28, examines in considerable detail the various forms of self-government and discipline adopted by the vocational organisation, in their origins, lines of progress, and relationship to the needs of the community at large. Though it is impossible within the compass of a brief article to deal adequately with the many points of interest raised in the section devoted to the medical profession, one may perhaps touch upon some of the more salient conclusions and recommendations. Originally under the control of the ecclesiastical authorities, it was for more than three hundred years, from the beginning of the sixteenth right down to the middle of the nineteenth century, broken up into three separately organised and exclusive vocations, the physicians, the surgeons, and the apothecaries, or as they became later, the general practitioners.

### The Medical Reform Act.

The chaos existing before the passage of the Medical Act of 1858 seems inconceivable. There were no fewer than nine medical corporations, which granted diplomas in either medicine or surgery, and, in addition, six universities, each of which granted degrees in its medical faculty, authorising its graduates to practise as physicians within certain circumscribed areas. Each university or corporation exercised a certain jurisdiction over a particular geographical area, which not infrequently overlapped that under the control of another faculty, and, in addition, there worked practically unchecked a host of unqualified practitioners, herbalists, bonesetters, midwives, male and female, druggists and chemists, who were not apothecaries, and dealers in the supernatural, or, again, others possessing a high degree of skill, but who were not legally entitled to exercise their calling in a particular area. The profession was, moreover, divided against itself. The members of the Royal Colleges of Surgeons and Physicians sought by every means in their power to confine to themselves the right to prescribe medicines, and it was only after a long conflict between them and the Society of Apothecaries, the latter originally a mere City Company, that the Colleges had to relinquish their exclusive claims. These licentiates of the Society of Apothecaries could, if they wished, obtain a diploma from the College of Surgeons, and were then styled apothecary surgeons. It is they who in lineal descent have become the general practitioners of the present day.

The Medical Act of 1858 achieved the two main purposes of the reformers—the merging of the three separately organised and mutually exclusive vocations perpetually fighting one another, physicians, surgeons, and apothecaries, into one united medical profession, and the enforcement of a uniform minimum standard of examination upon the licensing authorities. But no attempt was made to suppress quacks, other than the very effective regulation prohibiting them from suing for fees in a court of law, signing a death certificate, or exercising any public function of a medical character; a device which, apart from a few scattered bonesetters and faith-healers, has proved effective in safeguarding the confines of the medical profession. The General Medical Council, composed of thirty-eight members, all of whom are medical men (though there is no reason why some, or all, should not be laymen), is now the supreme governing body of the medical profession, numbering some 40,000 registered practitioners, in respect of training and discipline. The functions of the Council are twofold, to advise the Privy Council what qualifications shall be accepted as registrable, and to keep a list of medical men so registered. It possesses, moreover, the right of removing a practitioner from the Register, either for having been convicted of a felony in the civil courts, or for "infamous conduct in a professional respect."

But gradually the General Medical Council has assumed what are virtually legislative functions, it has formulated certain rules of professional conduct, and issues from time to time warning notices to the effect that any practitioner committing a breach of professional etiquette in a number of specified ways renders himself liable to be struck off the Register.

### The British Medical Association.

But, in spite of the dignity and great importance of the Medical Council, a number of subsidiary Associations have sprung up within the profession, the chief of which is naturally the British Medical Association. Founded in 1833 as the Provincial Medical Association, it gradually increased its influence, till in 1856 it migrated to London, founded the *British Medical Journal*, and became the British Medical Association. At present its members number fully half the profession, and their influence upon medical policy is immense. It was by their agency and the skilful use of that efficient weapon of the manual worker, a threatened strike of their members, that they were able to obtain, at the time of the passing of the Insurance Act, a further million and a half of public money over and above the sum Mr. Lloyd George had set aside for their payment, as well as certain other concessions, such as the "free choice of doctors," freedom from the control of the Friendly Societies, etc. It is notable that the most effective methods of industrial organisations for their own betterment, the strike, the boycott, and the blacklisting of undesirable and underpaid appointments, have been copied with much success by this Association. "The blacklisting of appointments," says the Report, "has been successful in many instances; and the governors of hospitals, the executive committees of Friendly Societies, and local authorities have all been obliged to offer better terms in particular cases. Besides fighting individual cases of sweating, the British Medical Association has laid down a scale of minimum salaries for public appointments, and has insisted that women practitioners must be paid the same as men for the same work.

Of medical ethics, the Report, in pointing out that one medical man is forbidden to offer any criticism of another, remarks that such a "prohibition seems sometimes to conflict with the best interests of the community at large."

Whole-time Appointments.

From the standpoint of the Fabian Society, there is naturally most room for criticism in the consideration of the relations between the medical practitioner and those whom he serves; in economic language the producer on the one hand and the consumer on the other. "Vocational organisation . . . has always clung to what is the heart's desire of every practitioner—namely, that a doctor should deal only with individual clients, in a strictly personal relation, without inspection or supervision, and free from outside control other than the general rules of conduct that might be made by his own professional organisation. Thus the doctors have always objected to anything in the nature of service under an Association of Consumers, whether Governmental or voluntary, not merely because they prefer remuneration by fees charged to individual clients rather than any commutation in the form of capitation fees, or even a fixed retainer or whole-time salary, but chiefly because service under an Association of Consumers leads to (*a*) supervision or inspection, or authoritative criticism, even if professional, of the work of the individual practitioner; (*b*) systematic allocation of work, either by grades of difficulty or kinds of service; and (*c*) the assumption of control by a lay committee, whether representative of the patients, of the subscribers, of the taxpayers, or of the State. . . . The sudden and quite unparalleled increase in whole-time medical appointments which has been caused by the war, and the consequent dislocation of particular private practices, is leading many doctors to speculate whether, in the social reconstruction that must follow the war, medicine, like teaching, may not become predominantly a public service, carried out in the main by salaried practitioners placed where they are most required." There is, the Report affirms, in the world of medical men, as in the world of industrial workers, an instinctive hostility for what is termed, in short, "scientific management." "This insistence that the workman shall at all times use his muscles in the exact way that results in the maximum production, instead of in the way that he chooses, seems to the wage-earner incompatible with personal freedom." It is unfortunately impossible here to enter into the many arguments adduced in favour of a State Medical Service, nor to consider the allegations of narrowness, of trade unionism in its worst form, which are brought against the British Medical Association.

Qualifications for Hospital Staffs.

But one more quotation must be given. "The monopoly of hospital appointments maintained by the Fellows of the Royal Colleges of Physicians and Surgeons, and the influence exercised by the Councils of these corporations, is, we think, in no small degree responsible for the concentration of hospitals in those towns and those parts of great cities which are convenient of access to these leading physicians and surgeons, instead of hospitals being placed where required by the population. The interests of holders of hospital appointments and not the interests of the patients and the public have been served by the general hospital policy of providing in the main for the classes of cases of the greatest interest to the medical profession rather than those of infectious fevers, "chronics," maternity, infancy, childhood, and convalescence. And, in so far as the administration of the voluntary hospitals is open to criticism, we cannot avoid being struck by the opposition maintained by the profession to any administration other than that which the surgeons and physicians may incidentally exercise."

Statements so unjust as these ought not to pass unchallenged.

